# Characterization of the complete chloroplast genome of *Sargassum fusiforme* and its phylogenomic position within phaeophyceae

**DOI:** 10.1080/23802359.2019.1671246

**Published:** 2019-09-26

**Authors:** Yonghua Zhang, Shengqin Wang, Weiguo Qian, Yijian Shi, Nan Li, Xiufeng Yan, Huixi Zou, Mingjiang Wu

**Affiliations:** a College of Life and Environmental Sciences, Wenzhou University, Wenzhou, China;; b Key Laboratory of Saline-Alkali Vegetation Ecology Restoration, Ministry of Education (Northeast Forestry University), Harbin, China;; c College of Electrical and Electronic Engineering, Wenzhou University, Wenzhou, China

**Keywords:** *Sargassum fusiforme*, PacBio sequencing, plastome, phylogenomics

## Abstract

*Sargassum fusiforme* is an important economic seaweed in East Asia. In this study, we characterized the complete chloroplast genome sequence of *S. fusiforme* using PacBio long-read sequencing technology. It had a circular mapping molecular with the length of 124,286 bp, with a large single-copy region (LSC, 73,437 bp) and a small single copy region (SSC, 40,131 bp) separated by a pair of inverted repeats (IRs, 5,359 bp). The cp genome contained 173 genes including 139 protein-coding, 6 rRNA, and 28 tRNA genes. The phylogenomic analysis indicated that *S. fusiforme* is closely related to *S. thunbergii*.

*Sargassum fusiforme* (Harvey) Setchell (= *Hizikia fusiformis* (Harvey) Okamura), widely applied as food, polysaccharide source, and medical agent, is a perennial brown macroalga distributed in the lower intertidal zones along the west of northern Pacific (Yu et al. 2012). Despite the importance of the species, there has been no genomic studies on *S. fusiforme* except the study of its mitochondrial genome (Liu et al. 2016). In this study, we reported and characterized the complete chloroplast genome of *S. fusiforme* using PacBio long-read sequencing technology. Moreover, the phylogeny of Phaeophyceae was reconstructed by utilizing the published related species’ chloroplast genome sequences to reassess the reliable taxonomic status of *S. fusiforme*.

One *S. fusiforme* individual was collected from Dongtou Island, Zhejiang Province of China (27°48′50′′ N, 121°10′1′′ E), and stored at Wenzhou university at −80 °C for DNAs isolation. Its voucher specimen was deposited in the Herbarium of Wenzhou University (Qian, qwg141101). Dozens of leaves (vesicle) were used to extract the total genomic DNA of *S. fusiforme* by using a plant genomic DNA extraction kit (Annoroad Gene Technology, Beijing, China). The chloroplast genome was reconstructed based on the PacBio Sequel data. Approximately 13-Gb sequence data were randomly extracted from the total sequencing output and used as input for Organelle_PBA (Soorni et al. 2017) to assemble the chloroplast genome. The chloroplast genome of *S. thunbergii* (GenBank accession number: NC_029134) was used as the reference sequence. The Illumina HiSeq data were used to correct the assembled genome using the Pilon software program (Walker et al. 2014). Gene annotation was conducted via the online program Dual Organellar Genome Annotator (DOGMA; Wyman et al. 2004) using the same method as Liu, Li, Worth, et al. (2017) and Liu, Li, et al. (2018).

The complete cp genome of *S. fusiforme* (GenBank accession number: MN121852) was 124,286 bp long comprising a pair of inverted repeat regions (IRs with 5,359 bp) divided by two single-copy regions (LSC with 73,437 bp and SSC with 40,131 bp). The overall GC content of the total length, LSC, SSC, and IR regions were 30.4%, 59.1%, 32.3%, and 8.6%, respectively. The cp genome encoded a total of 173 genes, of which 168 were unique and 5 were duplicated in the IR regions. The 173 unique genes consisted of 139 protein-coding genes, 28 tRNA genes, and 6 rRNA genes.

Maximum-likelihood (ML) analyses were run on a data set that included 126 protein-coding genes for 13 taxa in Phaeophyceae using RAxML v. 8.2.12 on CIPRES (http://www.phylo.org) under the GTR model. Our phylogenomic tree showed a good resolution of the species of Sargassaceae and other families in Phaeophyceae, with full support for all the nodes. The phylogenomic result (Figure 1) is consistent with the prior phylogenetic study on the taxonomic status of *S. fusiforme* (Stiger et al. 2003; Liu, Pang, et al. 2016; Liu, Pan, et al. 2018) confirming that *S. fusiforme* should not be assigned to the distinct genus *Hizikia*. The phylogenomic analysis showed that *S. fusiforme* is closely related to *S. thunbergii*.

**Figure 1.**
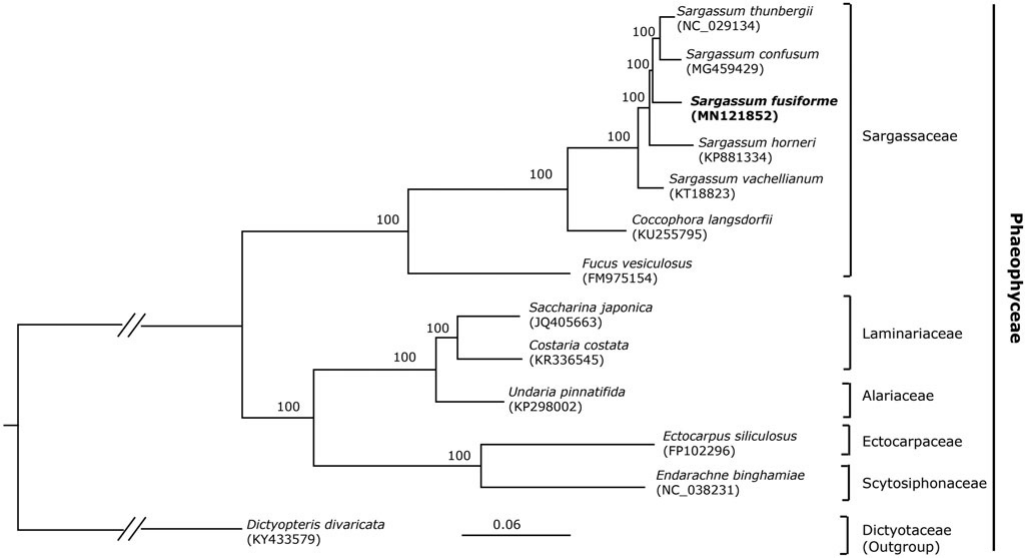
Phylogenomic tree reconstruction of 13 taxa of Phaeophyceae using ML method. Relative branch lengths are indicated. Numbers near the nodes represent ML bootstrap value.

## References

[CIT0001] Liu LX, Li R, Worth JRP, Li X, Li P, Cameron KM, Fu CX 2017 The complete chloroplast genome of Chinese bayberry (*Morella rubra*, Myricaceae): implications for understanding the evolution of Fagales. Front Plant Sci. 8:968. 28713393 10.3389/fpls.2017.00968 PMC5492642

[CIT0002] Liu LX, Li P, Zhang HW, Worth J 2018 Whole chloroplast genome sequences of the Japanese hemlocks, *Tsuga diversifolia* and *T. sieboldii*, and development of chloroplast microsatellite markers applicable to East Asian *Tsuga*. J Forest Res. 23:318–323.

[CIT0003] Liu F, Pan J, Zhang ZS, Moejes FW 2018 Organelle genomes of *Sargassum confusum* (Fucales, Phaeophyceae): mtDNA vs cpDNA. J Appl Phycol. 30:2715–2722.

[CIT0004] Liu F, Pang SJ, Luo MB 2016 Complete mitochondrial genome of the brown alga *Sargassum fusiforme* (Sargassaceae, Phaeophyceae): genome architecture and taxonomic consideration. Mitochondrial DNA. 27:1158–1160. 24989050 10.3109/19401736.2014.936417

[CIT0005] Soorni A, Haak D, Zaitlin D, Bombarely A 2017 Organelle_PBA, a pipeline for assembling chloroplast and mitochondrial genomes from PacBio DNA sequencing data. BMC Genomics. 18:49. 28061749 10.1186/s12864-016-3412-9 PMC5219736

[CIT0006] Stiger V, Horiguchi T, Yoshida T, Coleman AW, Masuda M 2003 Phylogenetic relationships within the genus *Sargassum* (Fucales, Phaeophyceae), inferred from ITS-2 nrDNA, with an emphasis on the taxonomic subdivision of the genus. Phycological Res. 51:1–10.

[CIT0007] Walker BJ, Abeel T, Shea T, Priest M, Abouelliel A, Sakthikumar S, Cuomo CA, Zeng Q, Wortman J, Young SK, et al. 2014 Pilon: an integrated tool for comprehensive microbial variant detection and genome assembly improvement. PLos One. 9:e112963. 25409509 10.1371/journal.pone.0112963 PMC4237348

[CIT0008] Wyman SK, Jansen RK, Boore JL 2004 Automatic annotation of organellar genomes with DOGMA. Bioinformatics. 20:3252–3255. 15180927 10.1093/bioinformatics/bth352

[CIT0009] Yu SH, Deng YY, Yao JT, Li SY, Xin X, Duan DL 2012 Population genetics of wild *Hizikia fusiformis* (Sargassaceae, Phaeophyta) along China’s coast. J Appl Phycol. 24:1287–1294.

